# Strength, Struggles, and Scholarly Aspirations: An Innovative Use of SWOT Analysis to Enhance Academic Productivity Among Pediatric Hospitalists

**DOI:** 10.7759/cureus.102828

**Published:** 2026-02-02

**Authors:** Katherine O Salada, Emily Jacobson, Rebekah Shaw, Derek Spindler, Christine Mikesell, Zarina Norton

**Affiliations:** 1 Department of Pediatrics, C. S. Mott Children’s Hospital, University of Michigan, Ann Arbor, USA; 2 Division of Hospital Medicine, Department of Internal Medicine, Michigan Medicine, University of Michigan, Ann Arbor, USA; 3 Division of Pediatric Hospital Medicine, Department of Pediatrics, C. S. Mott Children’s Hospital, University of Michigan, Ann Arbor, USA

**Keywords:** academic productivity, career development, pediatric hospital medicine, professional development, swot analysis

## Abstract

Background

The Strengths, Weaknesses, Opportunities, and Threats (SWOT) strategy is widely recognized in business but underutilized in academic medicine. This project applied SWOT analysis to assess academic success and productivity within the pediatric hospital medicine (PHM) division at a large quaternary children’s hospital.

Methodology

This qualitative study included nine faculty members from the PHM division at C. S. Mott Children’s Hospital, Ann Arbor, Michigan, in April 2025. An interactive workshop on scholarly productivity was held, using prompts to facilitate discussion of SWOT. Written responses were collected, transcribed, and aggregated for analysis. Three investigators collaboratively coded data until thematic saturation was reached, using inductive thematic analysis to identify themes. Member checking and peer debriefing were completed to ensure the validity of the results.

Results

Nine PHM faculty attended the workshop. Division strengths included clinical excellence, diverse expertise, leadership, and collaboration. Weaknesses included limited academic knowledge, resources, time, competing demands, and limited academic self-efficacy. Opportunities centered on systems-based work, clinical scholarship, and professional growth. Threats included risks to promotion, compensation, professional fulfillment, recognition, and work balance. Findings guided requests for departmental support, new divisional goals, and identified potential academic projects.

Conclusions

This project demonstrates the rapid application of SWOT analysis within an academic division, offering a replicable framework for other institutions to assess needs, guide targeted resource allocation, and foster collaboration to improve academic productivity.

## Introduction

The role of academic pediatric hospitalists has evolved since the inception of hospital medicine in the 1990s [1] and the subsequent designation of pediatric hospital medicine (PHM) as a distinct subspecialty in 2016 [2]. Pediatric hospitalists are uniquely positioned to lead institutional, regional, and national initiatives to improve high-value hospital care quality and efficiency [3]. To meet these aims, hospitalists must engage in scholarly activity; however, many PHM faculty are not fellowship trained and lack significant research experience [4-6]. For example, one study found that 60% of academic adult hospitalists have not presented a poster at a national meeting and 50% have not authored an academic paper [7]. Potential barriers to academic productivity, extrapolated from studies of adult hospital medicine and emergency medicine providers, include insufficient protected time due to primarily clinical roles, inadequate infrastructure, and a lack of mentorship [8,9,10]. Despite these barriers, PHM faculty and programs will likely continue to place a high value on scholarship, given its impact on care quality, fellowship program success, and potential implications for career development and promotion at academic institutions. However, there are limited data on strategies to improve scholarly productivity among PHM physicians, both nationally and within our own institution.

The Strengths, Weaknesses, Opportunities, Threats (SWOT) analysis was originally developed as a business strategy tool. More recently, several studies have emerged using the SWOT framework as a rapid analysis tool for program evaluation and improvement in the healthcare setting [11,12]. For instance, pediatric anesthesia teams have employed SWOT analyses to systematically identify both internal and external challenges [12], while rehabilitation teams have leveraged the tool to reorganize patient care models [11]. These examples highlight the potential utility of SWOT analysis in healthcare - not only for illuminating the current operational landscape, but also for pinpointing areas for improvement and aligning strategic objectives. Although other quality improvement tools exist, such as key driver diagrams, value stream mapping, and failure modes and effects analysis, these methodologies typically excel at targeting and achieving specific outcomes. In contrast, SWOT analysis provides a broader strategic overview, serving as a foundation upon which targeted quality improvement initiatives can be built. By first using the SWOT framework to rapidly capture the wide-ranging dimensions of a program’s current state, subsequent tools can complement one another and drive more focused improvements. This study aimed to utilize the SWOT framework to facilitate a qualitative study to identify areas of strengths, opportunities, barriers, and threats related to academic productivity and scholarship within an academic PHM division.

## Materials and methods

This qualitative study was conducted in April 2025 with faculty members from the PHM division at C. S. Mott Children’s Hospital in Ann Arbor, Michigan, a quaternary care children’s hospital. The division includes 39 faculty members, 10 of whom have completed a pediatric subspecialty fellowship (7 in PHM, 2 in infectious diseases, and 1 in critical care medicine). A PHM fellowship has been established at the site since 2021.

To identify and address perceived gaps in scholarly productivity, study investigators planned a 30-minute interactive workshop during the division’s annual retreat. A facilitation guide was developed by two local PHM Research Group leaders (Appendix), each with medical education and qualitative research experience. The guide was structured around the established SWOT framework. As no validated facilitation guide existed for this context, face validity was established through expert consensus and review within the research group.

All participants provided informed consent before participating. This study was deemed exempt by the Michigan Medicine institutional review board. During the annual in-person retreat, faculty could opt in to various sessions, including this session, resulting in a self-selection sample with possible implications for generalizability.

The session was facilitated by two PHM faculty members and began with an introduction to the project, a description of the SWOT workshop, and a brief demographic survey. Next, participants were given writing material and led through structured prompts in each SWOT category, specific to scholarly productivity. Each category began with a 60-second activity during which participants independently generated ideas in timed writing intervals, recording one idea per note page in response to a given prompt. Notes were then collected in one pooled sample and sorted by concept theme onto a central board, followed by a facilitated group discussion based on the topics identified. A running list of potential scholarship projects was created throughout the session.

Following the workshop, all responses were transcribed into a spreadsheet. Three investigators collaboratively performed inductive thematic analysis to identify themes and sub-themes in real time. Coders began with initial data familiarization, followed by group review and assignment of preliminary codes via consensus. A shared codebook was developed and refined iteratively. After all responses were coded, they were organized into themes and sub-themes, which were reviewed by the entire study team and refined to ensure they accurately reflected the participants’ perspectives and maintained fidelity. As codes were reviewed and themes identified, it was evident that no new themes were emerging, and thematic saturation was reached. The study team conducted member checking at several points, including during initial workshop discussion, during coding, and when refining themes. Peer debriefing was completed by discussing themes with peers (PHM colleagues not involved in the study) to help mitigate bias and ensure colleagues outside the study corroborated the identified themes. Investigators regularly practiced reflexivity during the thematic analysis process.

## Results

Nine PHM faculty (23% of the division) participated in the SWOT workshop, with a median of four years of experience (interquartile range (IQR) = 2.7), and the majority (6, 67%) had no fellowship training. While most had previously presented their scholarly work locally (7, 78%), nearly half (4, 44%) lacked prior regional or national scholarly presentations. All participants reported prior scholarly publication(s).

Using thematic analysis, the SWOT framework identified key themes affecting academic productivity within the division (Table 1). Personal and division strengths included (1) excellence in clinical care; (2) breadth and depth of academic interests, expertise, and leadership; and (3) strong collaboration and communication. Weaknesses highlighted (1) insufficient infrastructure and resources, (2) limited academic self-efficacy, (3) insufficient protected time for scholarly activity, and (4) gaps in knowledge and skill development. Opportunities focused on (1) advancing system-level improvement through scholarly work; (2) advancing clinical excellence through scholarship; and (3) pathways to professional growth and academic development. Finally, faculty identified key threats as (1) stalled academic promotions and reduced salary; (2) reduced professional fulfillment and engagement; (3) reduced recognition regionally and nationally; and (4) concerned about an unsustainable imbalance between clinical and academic work.

**Table 1:** Thematic analysis. *Due to the nature of this rapid workshop event, participants were intentionally prompted to write numerous quick notes in a timed session, resulting in short/partial quotes that at times included phrases or single words. SWOT, strengths, weaknesses, opportunities, threats

SWOT category	Themes	Subthemes	Examples*
Strengths	Excellence in clinical care	(1) Expertise in complex care and care coordination	“Clinical care” “Care of hospitalized children” “Clinical care of complex patients” “Complex care coordination”
	Breadth and depth of academic interests, expertise, and leadership	(1) Medical education, (2) Quality improvement and hospital operations	“Medical education” “Quality improvement” “Innovation” “Leadership roles” “Diversity of interests” “Guideline development”
	Strong culture of collaboration and communication		“Collaboration” “Communication” “Multidisciplinary care”
Weaknesses	Gaps in knowledge and skill development		“Knowing where to publish,” “Knowing who else is interested (to help),” “Methods knowledge.”
	Insufficient infrastructure and resources		“Money” “Statistical support” “Lack of resources (stats, time, other jobs)” “Assistance with data pull”
	Insufficient protected time for scholarship		“Competing demands” “Time” “Protected time” “Time for data collection”
	Limited academic self-efficacy		“Imposter syndrome” “Big first step - intimidating” “View of department that we are not scholarly”
Opportunities	Advancing system-level improvements through scholarly work	(1) Improving hospital throughput and patient flow, (2) Reducing low-value or unnecessary care, (3) Enhancing communications across teams and settings	“Decrease monitors” “Decrease secure chats” “Documentation” “Complex discharges (need checklist)” “Waiting for sedated procedures.”
	Advancing clinical excellence through scholarly work	(1) Enhancing care delivery in the newborn setting, (2) leadership in antibiotic stewardship	“De-escalating antibiotics,” “MRSA swabs to decide on Vancomycin,” “Orbital cellulitis clinical practice guideline,” “Newborn refusals,” “Weaning fluids vs just stopping them.”
	Pathways to professional growth and academic development		“Mentorship” “Engage learners” “Collaboration (nationally and within institution)” “Think about making work scholarly.”
Threats	Stalled academic promotion and salary implications		“No promotion (and no increase in salary)” “Failure to promote” “Lower salary/money”
	Reduced professional fulfillment and engagement		“Erosion of identity,” “Burnout,” “Lack of personal/professional fulfillment”
	Decreased recognition and scholarly visibility		“Decreased national recognition for institution” “Lack of regional/national presence”
	Unsustainable imbalance between clinical and academic work		“More clinical time threatens research time.” “Decreased protected time/increased clinical time furthering the problem.” “Decreased scholarship opportunities”

This analysis allowed our division to identify actionable needs and next steps for improving academic productivity. The strengths and opportunities were channeled into project and networking ideas for scholarly activity and collaboration, with a list of potential scholarly projects drafted during the session. The discussion surrounding weaknesses and barriers was utilized to develop a concrete list of needs from our institution, including protected time for scholarly activity, resources such as statistical support, and more structured education and mentorship. We clearly conveyed the threats our division faces if scholarly productivity does not improve, including reduced professional fulfillment and advancement.

## Discussion

This study demonstrates that SWOT analysis is a practical and efficient method for identifying barriers and facilitators to academic productivity within a pediatric hospital medicine division. By organizing discussions around strengths, weaknesses, opportunities, and threats, the group developed a comprehensive understanding of the division’s current landscape and articulated specific requests for institutional support. Locally, these insights empower our division to advocate for increased resources, design targeted scholarly initiatives, and collaborate with leadership to secure programmatic support that will both sustain and elevate academic output. Moving forward, these priorities will be further examined using complementary quality improvement tools, such as key driver diagrams, to more precisely link identified factors to actionable interventions. Importantly, our findings align with Bland’s model of faculty productivity, which recognizes the dynamic interplay of individual, organization, and leadership factors in promoting academic achievement [13].

As clinical and administrative pressures on PHM faculty increase, the value of a structured, rapid analysis tool becomes ever more important [5,14]. Our study adds to the emerging body of literature describing opportunities for implementation of the SWOT tool in healthcare, and future validation of the SWOT tool in this setting would improve awareness and accessibility of this tool. Potential implications for this tool extend well beyond assessing divisional productivity needs, with potential applications including assessing a wide array of clinical, operational, scholarly, administrative, and medical education needs for the local, regional, and national medical community at large.

Limitations

While many of these factors will be similar between institutions, PHM divisions across the country face unique strengths, barriers, and opportunities to increase scholarship. Although this workshop was conducted within a single academic division and the projects’ small sample size limits direct generalizability, the SWOT framework itself is adaptable and can be tailored by other institutions to their unique contexts and needs. Furthermore, by designing this study as an interactive workshop, we were unable to directly compare the SWOT framework to other well-known quality improvement or implementation frameworks. Our goal is to describe the novel use of the SWOT tool, which is more generalizable, using our group’s results as an example. Further studies would benefit from establishing the validity of the SWOT tool in healthcare settings, analyzing the SWOT framework in comparison with other rapid improvement tools, and understanding how these different tools may complement one another.

Participation was via convenience sampling (self-selection) rather than purposive sampling, although there was a distribution of key demographics in our participants (e.g., years as faculty, number of previous scholarly products), which we would have aimed for in a purposive sample. Faculty self-selected to participate in a small group focused on scholarly activity and thus may have been biased toward a greater value placed on academic scholarship compared to non-participants. Conversely, participating faculty may have a deeper understanding of factors related to academic productivity, contributing to more robust results and greater insights. Peer debriefing ensured that identified themes resonated with colleagues not involved in the study. While individuals generated ideas and thoughts independently at first, discussion may have been biased by group input. We limited time spent on each prompt to complete all aspects of the SWOT analysis; however, participants may have identified additional ideas if allotted additional time. Finally, while generated ideas led to concrete asks from our institutional leadership and specific opportunities for scholarly work, it remains uncertain whether these ideas will lead to action or improve scholarly productivity.

## Conclusions

While our thematic analysis reflects the specific needs of our local division, this exploratory project adds to the growing body of evidence, suggesting that the SWOT framework has potential to serve as a more broadly applicable model that can be applied to support other institutions in their efforts to identify division needs and seize opportunities for scholarly and operational advancement. The example workshop presented here may serve as a framework for other institutions to analyze their division needs, allowing for targeted resource allocation, identification of unique opportunities for scholarship utilizing known strengths, and recognition of threats if goals are not met. Future work should focus on broader validation of the tool and tracking its impact on academic outcomes.

## Figures and Tables

**Figure 1:**
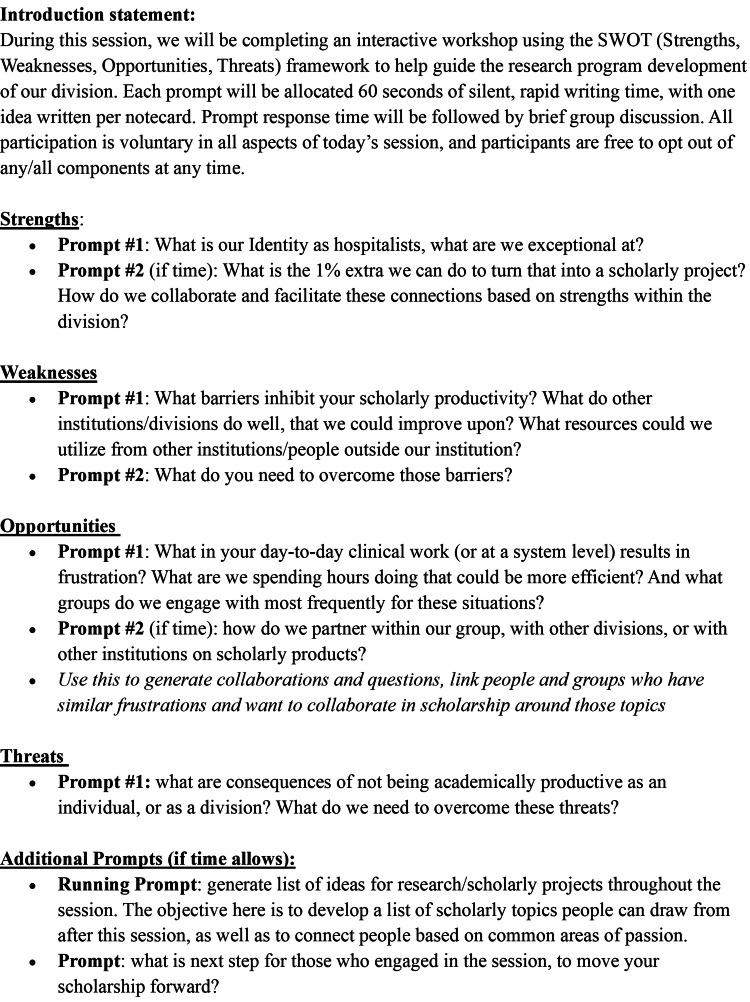
Workshop Facilitation Guide.

## Appendices

Appendix 
